# Mining textural knowledge in biological images: Applications, methods and trends

**DOI:** 10.1016/j.csbj.2016.11.002

**Published:** 2016-11-24

**Authors:** Santa Di Cataldo, Elisa Ficarra

**Affiliations:** Dept. of Computer and Control Engineering, Politecnico di Torino, Cso Duca degli Abruzzi 24, Torino 10129, Italy

**Keywords:** Textural analysis, Bioimaging, Textural features extraction, Texture classification, Feature encoding, Deep learning

## Abstract

Texture analysis is a major task in many areas of computer vision and pattern recognition, including biological imaging. Indeed, visual textures can be exploited to distinguish specific tissues or cells in a biological sample, to highlight chemical reactions between molecules, as well as to detect subcellular patterns that can be evidence of certain pathologies. This makes automated texture analysis fundamental in many applications of biomedicine, such as the accurate detection and grading of multiple types of cancer, the differential diagnosis of autoimmune diseases, or the study of physiological processes. Due to their specific characteristics and challenges, the design of texture analysis systems for biological images has attracted ever-growing attention in the last few years. In this paper, we perform a critical review of this important topic. First, we provide a general definition of texture analysis and discuss its role in the context of bioimaging, with examples of applications from the recent literature. Then, we review the main approaches to automated texture analysis, with special attention to the methods of feature extraction and encoding that can be successfully applied to microscopy images of cells or tissues. Our aim is to provide an overview of the state of the art, as well as a glimpse into the latest and future trends of research in this area.

## 1 Texture analysis: definition and main application areas

**Fig. 1:**
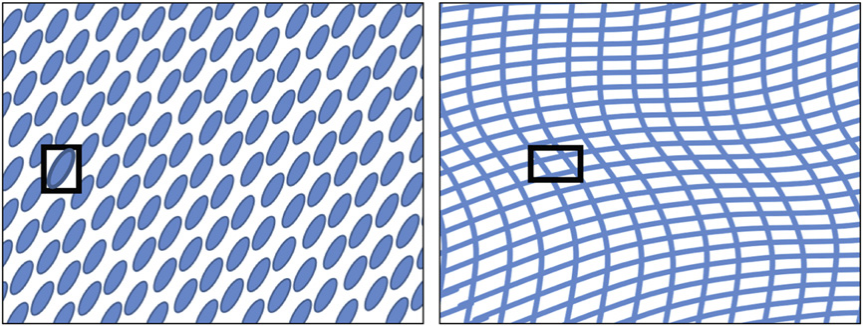
Visual textures with corresponding subpatterns.

Texture analysis attempts at the formalisation of an inherently informal concept, that is the appearance and feel of visual textures in an image. Generally speaking, visual textures are nonrandom arrangement of entities (*subpatterns* [1]) with a certain distribution of brightness, colours, shapes, etc. (see Fig. 1). The fine aggregation of the subpatterns in the observer's eye generates the perception of texture as a whole, even in absence of well-defined boundaries.

Texture analysis has received attention from the research community since the early 70s, and over the years it has been successfully applied to a large number of tasks in computer vision. Among the others:

– *Image segmentation.* Leveraging on the variation of textures with respect to the background, it is possible to identify objects or regions of interest, even though their boundaries are poorly defined or non-existent. For example, a traditional application in computer vision is the segmentation of natural scene images, especially from remote sensing devices [2], [3].
– *Object classification.* Textural characteristics allow to infer physical or chemical properties of the imaged objects. This allows, for example, to classify objects' materials [4] or, in case of medical images, to categorise a patient into a specific range of diseases [5].
– *Image and video compression.* Robust texture representations are essential to achieve efficient and loss-less compressions of digital images [6].
– *Content-based image retrieval.* Texture descriptors provide compact characterisations of the image content, allowing the automatic retrieval of images from databases without need of metadata indexing [7].
– *3D scene reconstruction and rendering.* 3D shape information about objects can be inferred from two-dimensional texture using cameras from specific viewpoints (*shape-from-texture* [8], [9]).

The perception and segregation of different textures in an image has much to do with the way the visual patterns are processed and aggregated by the human visual cortex. Even for the simplest forms of textures, the formalisation of this process into compact mathematical definitions can be very challenging, and may require a-priori assumptions about the distribution of intensities in subregions of the image. Such assumptions are unavoidably context-specific, as they depend on the unique characteristics of the targeted images.

General approaches of texture analysis can be shared among different applications and types of images. Nevertheless, specific imaging contexts, such as bioimage processing, need textural descriptors able to reflect their peculiar characteristics and challenges [10].

In this paper, we will go deeper into the role and fundamentals of textural analysis in bioimage informatics.

## 2 Texture analysis in biological imaging

The automated analysis of textures has always been a topic of importance in biomedical imaging and especially in the radiology sector, involving different imaging techniques such as X-ray radiography, ultrasound (US), computed tomography (CT), positron emission tomography (PET) and magnetic resonance imaging (MRI) [11]. Due to its superior characteristics in terms of image definition and soft tissues discrimination capabilities, MRI is by far the one where texture analysis has found the highest variety of applications, which include the segmentation of different anatomical areas, the differentiation between normal and pathological subjects as well as the classification and grading of a large number of pathological conditions. For example, widely referenced studies on brain MRI leverage on automated texture analysis to segment the cerebellum, the hippocampus or the corpus callosum, to aid the automated diagnosis of encephalopathy, multiple sclerosis or Alzheimer's disease, as well as to classify hippocampal alterations into different grades [12].

While the automated analysis of textures in medical images (e.g. MRI) has a quite consolidated tradition, microscopy-based bioimaging is a context where the human evaluation of the images has prevailed for a long time. Indeed, the interpretation of the biological specimens is traditionally considered a very complicated task, requiring experienced and well-trained operators. This complication is a consequence of the extreme variability affecting the images, where a “biological” noise, due to different types of cells and corpuscles of variable morphology coexisting in the same specimens, is added to a “technological” noise, due to the general lack of standards in the image generation and acquisition process [13].

Nonetheless, the considerable technological advance of microscopy and the increased availability of computational power at a lower cost have recently determined a growing interest of pathologists and biotechnologists for quantitative analysis systems, where the interpretation of the biological specimens is not left to the subjective evaluation of a human operator but based on analytic features automatically extracted from the digital images [14]. The reason of this interest is two-fold. First, higher accuracy and repeatability of the analysis' outcome. Second, reduced need for highly specialised operators, and hence much lower costs for the health system [15]. Hence, in the last few years the automated analysis of biological textures has become increasingly popular among computer scientists.

**Fig. 2:**
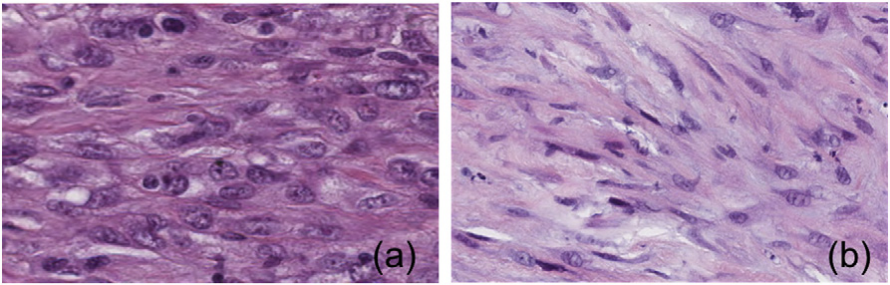
Different textures in H&E pulmonary tissues: (a) Sarcomatoid mesothelioma (cancerous). (b) Active fibrosis (non-cancerous).

**Fig. 3:**
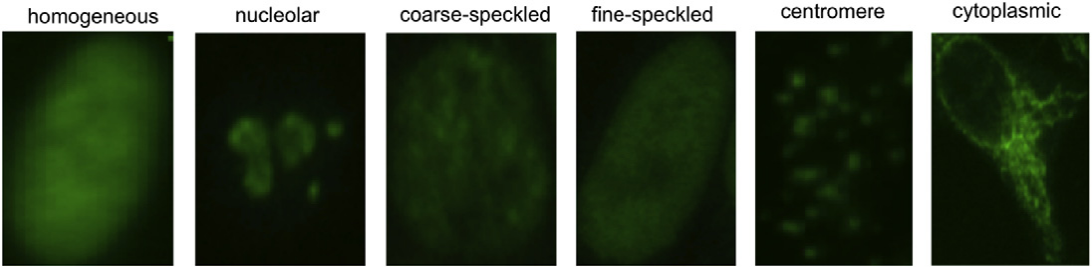
Textures categories in HEp-2 cell images for the differential diagnosis of autoimmune diseases.

In the biological images, we can call “texture” any special spatial arrangements of the biological components appearing in the image, which may have some relevance to a clinical or biological application. Depending on the scale of this spatial arrangement, we can roughly group these textures into two categories:

– In *tissue textures*, texture is a property of a tissue, or in general of a large area of the sample, and it is generated by a specific spatial arrangement of the cells in such area. In other words, the way the cells are positioned within the tissue have some kind of ordered structure, which can be defined as a texture (see two examples in Fig. 2).
– In *cell textures*, texture is a property of the individual cells. In this case, the special arrangement of the sub-cellular components (e.g. the nuclear chromatin) gives a well-recognisable pattern to the cells (see few examples in Fig. 3).

From a technological point of view, the spatial arrangement of the biological components is made visible to a microscope by chemical reactions between the biological sample and an external contrast agent, which are able to reveal specific cells or cellular parts of the specimen. Hence, the properties of the generated texture (spatial scale and colour of the patterns, noise level, etc.) depend not only on the type of cells/tissues, but also on the type of contrast agent and the chemical bond it exploits, which is characteristic of a specific microscope imaging technology.

In traditional light microscopy, the contrasting process is based on staining. For example, in Hematoxylin and Eosin histology (H&E, the most commonly used staining technique) Hematoxylin stains nuclei blue due to its affinity to the nucleic acids contained in the cell nucleus, while eosin, an acidic dye, stains the cytoplasm of the cells pink [16]. Thanks to the staining process, any nonrandom arrangements of the cells (cells more or less packed, with circular or elongated nuclei, disorganised or with a preferential direction, etc.) generate distinct blue & pink *tissue textures* in the biological image, as in Fig. 2.

The automated representation and classification of such textures can help in identifying specific tissues. This is exploited for a large number of useful purposes, including the segmentation of tissue areas, the discrimination between benign or malign lesions, as well as the identification and grading of cancers. For example, in Ref. [17], a large set of textural and nuclear architecture based features are extracted from H&E breast biopsy images. Then, automated classification based on support vector machines (SVM [18]) is used to distinguish between cancerous and non-cancerous images and to categorise the former ones into different grades of cancer. In Ref. [19], colour texture features are extracted to perform the automated segmentation of H&E follicular lymphoma cells. In Ref. [20], automated texture analysis based on statistical descriptors is successfully applied to H&E stained liver sections of rats to automatically distinguish subjects with fibrosis.

Differently from traditional staining techniques, the imaging technologies leveraging on immunohistochemistry (IHC) are able to reveal textures at a much finer spatial scale, because they can highlight very small molecular complexes such as proteins, carbohydrates or lipids [21]. Hence, such images can be exploited not only for tissue texture but also for *cell texture* analysis. IHC techniques rely on antibodies conjugated to either enzymes, that can catalyse colour-producing reactions, or to fluorophores (i.e. immunofluorescence). The antibodies specifically bind the target antigens in the tissue sample and create an antibody-antigen bond can be revealed using fluorescence microscopy or confocal laser microscopy, allowing to discriminate sub-cellular textures with a good level of detail.

The automated analysis and classification of the sub-cellular textures from IHC images can be exploited to obtain a subtle categorisation of many cellular types, which is useful to several clinical purposes. For example, the automated classification of epithelial type-2 (HEp-2) *cell textures* in immunofluorescence imaging allows the differential diagnosis of a number of serious autoimmune diseases such as lupus, rheumatoid arthritis and scleroderma. This application, called antinuclear antibody (ANA) test, has recently attracted a lot of attention from the research community. The specific sub-cellular patterns revealed on the HEp-2 cells are a consequence of the presence in the patients' serum of specific antibodies that are held responsible for the diseases (see few examples in Fig. 3). The correct identification of the HEp-2 pattern helps identifying the type of antibody, hence it indirectly allows a differential diagnosis of the autoimmune disease. In the last few years, many researchers have exploited the analysis of HEp-2 textures to either perform the automated classification of HEp-2 patterns [22], [23], the automated segmentation of HEp-2 cells [24], [25] or the recognition of mitotic processes within the HEp-2 samples [26], [27], which are all important tasks in the ANA testing procedure.

Depending on the specific application and on the imaging technology, the characteristics of the tissue or cell patterns in terms of scale, colour distribution, contrast, signal-to-noise ratio may change considerably (see Fig. 2, Fig. 3 as examples). Nonetheless, all the applications share two major points, that effective automated texture analysis systems need to handle.

First, as a drawback of the microscopy imaging technology per se, images are subject to major sources of noise and artefacts. For example, in cyto/histological imaging, noise might originate from a specific staining of the background or of structures which are not the intended targets. In fluorescence microscopy, image degradations might derive from the bleaching of the fluorophores after exposure to light. In general, major variabilities might occur due to changes in the instrument setup, or due to unwanted contaminations of the biological samples.

Second, differently from artificial ones, biological objects are naturally subject to shape and size variability. This variability is considerably amplified in presence of pathological phenomena. For example, cancer is often characterised by uncontrolled and irregular cellular growth, which alters the natural cell arrangement of the tissues. As such, basic definitions of texture as the repetitive and ordered arrangement of well-defined sub-patterns simply do not hold in this context.

In the following, we will discuss the basics and major trends of texture analysis, with special regard to approaches and techniques for the classification and segmentation of textures in biological images.

## 3 The texture analysis framework

A classic framework for texture analysis consists of three main steps:

1. *Feature extraction*: a set of local texture descriptors are computed from patches of the input image (or a region of interest obtained by image segmentation) and concatenated into a feature vector.
2. *Feature coding* (optional): local descriptors are converted into a compact statistical representation based on a pre-defined coding structure or dictionary.
3. *Texture classification*: the texture features (either from step 1 or 2) are fed into a classifier, that categorises unlabelled images or regions of interest into a certain number of texture classes. The classification can either be supervised, leveraging on pre-labelled training samples, or unsupervised, where the texture classes are gathered from the analysis of the hidden structure of input data in the features space.

While most of the algorithms proposed for step 3 are machine learning approaches that are well-established in all areas of computer vision (e.g. Support Vector Machines [28], [29], boosting algorithms [30], [31], neural networks [32], [33], and random forest techniques [34], [35]), most of the efforts of the research community are directed towards designing suitable texture descriptors for specific biological applications. Indeed, literature suggests that a smart choice and encoding of the features is by far the most important aspect in obtaining a accurate texture discrimination [36].

In the following, we give an overview of texture feature extraction and coding with special regard to biological image applications, and provide just a few glances to the classification step. For this, the interested reader can refer to the surveys on machine learning published by Refs. [37], [38].

## 4 Texture feature extraction

### 4.1 Geometrical or structural methods

This category of approaches apply the basic definition of texture as a regular repetition of sub-patterns or primitives. Based on this concept, they first identify such primitives, also called *texture elements* (e.g. edges, Voronoi polygons, and blobs), and then compute either statistical or morphological descriptors assuming certain placement rules of the primitives [39]. For example, in Ref. [40] segmented regions and lines in confocal scanning laser microscopy images of fetal liver cells are interpreted as texture primitives and stored in a uniform data structure that reflects the arrangement of the chromatin in the cell nuclei.

The assumption of homogeneous placement of the primitives is a major limitation. While this hypothesis generally holds very well for artificial textures, it is most of the times disproved in biological images. Hence, this approach is mostly unsuccessful when applied to images of cells or tissues.

### 4.2 Statistical methods

Texture can be defined not only as a deterministic repetition of sub-patterns, but also as a non-deterministic spatial distribution of intensity values. This latter definition is at the base of statistical methods for texture analysis.

The spatial distribution of intensities related to texture can be mathematically represented by a set of first- or second-order statistics:

– *First-order statistics* relate to the likelihood of individual pixels having specific intensity values.
– *Second-order statistics* relate to the joint likelihood of two random pixels in the image having specific pairs of intensity values.

First order statistics are gathered from the normalised intensity histogram of the image, that is a version of the intensity histogram where the grey level occurrences are normalised in order to obtain an estimation of the probability density function of the intensities. To characterise the shape of the intensity distribution, and hence the texture of the image, a set of statistical descriptors such as mean, variance, skewness, kurtosis, energy, and entropy can be computed either from the global histogram or from local intensity histograms of image patches [41].

**Fig. 4:**
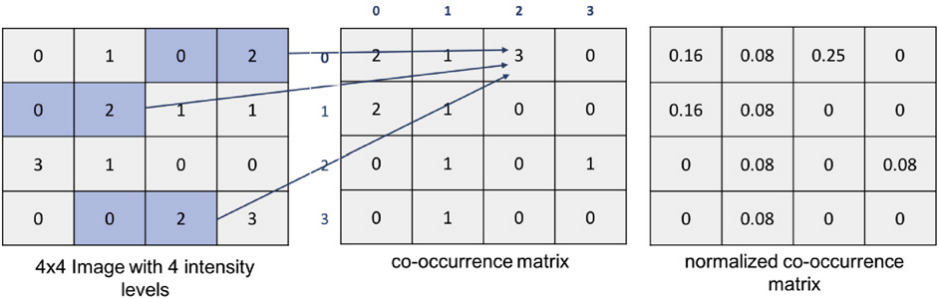
Computation of a normalised co-occurrence matrix with *d* = 1 and *θ* = 0.

While first order statistics have major advantages of simplicity and low computational cost, they are way too simple to characterise complex textures, hence they find little application to biological images. Much more attention is given to second order statistics, where the joint probability of pixel pairs are taken into account. They require to compute a second-order intensity histogram, the so-called *co-occurrence matrix* [42], that is a square matrix where each element in position (*i*,*j*) contains the probability for a pair of pixels located at a distance *d* and direction *θ* in the image to have intensity levels *i* and *j*, respectively (see example in Fig. 4). Starting from the normalised co-occurrence matrix, a number of texture descriptors can be computed such as angular second moment, contrast, homogeneity, entropy, and maximum joint probability.

Widely known studies on texture visual perception by a prominent visual neuroscientist, B.Julesz, showed that textures sharing the same second order statistics are not perceived as different by human observers, even if they have very different third order statistics [43], [44]. This suggests that second order descriptors might have the highest discriminative capability, even compared to higher order ones. Sure enough, as the computational complexity increases exponentially with the order of the statistics, second order descriptors are most of the times preferred in texture analysis literature [45].

On the other hand, this type of descriptor has two major limitations. First, the difficulty to set the orientation of the dipole (*d*,*θ*) in order to obtain optimal texture discrimination, which might be very image-dependent. Second, the lack of invariance of the obtained descriptors to size and rotation. Hence, rotated or scaled versions of the very same texture will be labelled differently, leading to classification errors. To partially overcome this problem, texture classification can be performed based on the mean and variance of second order statistics extracted for different values of *d* and *θ* [42]. Recent works also propose multi-scale extensions of the traditional co-occurrence matrix descriptors, based on combining features extracted from the entire matrix as well as from sub-windows [46].

### 4.3 Local binary patterns

**Fig. 5:**
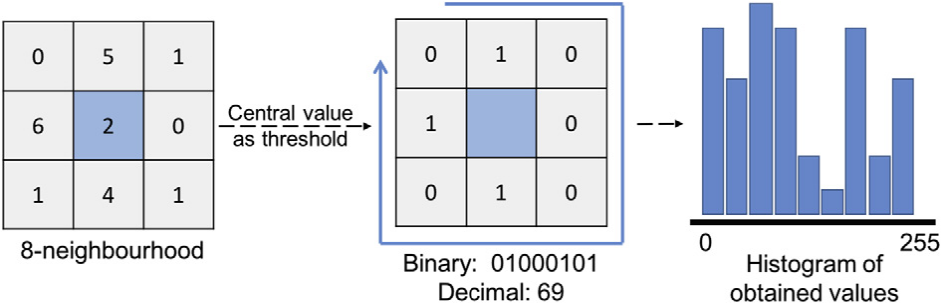
Computation of local binary patterns.

As a clever unification of structural and statistical texture analysis approaches, local binary patterns (LBP) were first proposed in 1994 [47], [48]. The basic idea behind this descriptor is to describe texture as a histogram of LBPs, i.e. binary patterns representing the intensity relations between a pixel and its neighbours. For each image pixel, a LBP is obtained by binarizing its neighbouring region using the intensity of the pixel as threshold, and then by converting the resulting binary pattern to a decimal number (see Fig. 5). Finally, a histogram is generated by taking into account the occurrences of all the LBPs in the image. This is a very simple yet powerful textural descriptor, whose main advantage is the invariance to changes of illumination over the image.

Recent literature proposes several variants of classical LPB formulation that are supposed to extend and improve its descriptive capabilities. Among the others:

– *Rotation-invariant uniform LBPs (*LBP*^*riu*2^)* Binary patterns are called *uniform* if they contain very less spatial transitions (i.e., no more than two bitwise 0/1 changes). As they contain fewer spatial transitions, uniform patterns are more tolerant to unwanted changes upon rotation. Hence, they are the most discriminative for characterising most textures. In Ref. [49], uniformity is exploited to generate compact rotation-invariant feature vectors.
– *Completed LBPs (CLBP)* In classical LBPs, all pixels are binarised using the central one as threshold. Hence, only the sign of the difference between the center and the neighbour grey values is relevant. Conversely, Completed Local Binary Patterns (CLBP [50]) represent each neighbourhood by its center pixel as well as by a local difference sign-magnitude transform (LDSMT). This way, they take into account both the sign and the magnitude of the difference between the central pixel and its neighbours.
– *Co-occurrence of Adjacent LBPs (CoALBP)* In the original expression of LBPs structural information among different binary patterns is missing. This is the idea behind the formulation proposed in Ref. [51], where the co-occurrence of multiple LBPs (and in particular, adjacent LBPs) is taken into account.
– *Rotation-Invariant Co-occurrence of Adjacent LBPs (RIC-LBP)* As CoALBP features are very dependent on the orientation of the target object, a work by Ref. [52] proposes a rotation invariant formulation. A rotation invariant label is attached to each LBP pair, so that all CoALBPs corresponding to different rotations of the same pattern are equivalent.
– *Globally rotation invariant multi-scale co-occurrence local binary pattern (MCLBP)* In MCLBPs, a smart encoding of local binary patterns is performed at multiple scales, in order to increase their discriminative capabilities [53]. All the co-occurrence patterns are arranged into groups according to properties of the co-patterns, and features are extracted from each group based on three different encoding strategies, designed to capture the correlation information between different scales and maintain rotation invariance.

Thanks to their advantages in terms of accurate and robust description of local information, LBPs have been successfully used to identify and classify biological textures in a number of important applications. For example, in Ref. [54] classical LBP and shape descriptors were used to classify lymphocyte cells and diagnose Acute Lymphoblastic Leukemia from optical microscopy images of blood samples. In Ref. [55], *LBP*^*riu*2^ features were used to detect candidate cells for apoptosis in phase-contrast microscopy images. In Ref. [22], CoALBP and RIC-LBP features applied to the classification of HEp-2 cell patterns for ANA testing outperformed a large number of other texture analysis methods applied to the same image datasets. In Ref. [56], a three-layered feature learning framework based on local binary patterns was successfully applied to protein classification in HeLa and Pap-smear fluorescence images.

On the other hand, the main disadvantage of LBPs is the computational burden of processing large number of features, especially for the most sophisticated formulations. Hence, several works suggest the use of feature reduction techniques such as Sequential Feature Selection [57] and Minimum Redundancy and Maximum Relevance (mRMR) algorithms [58].

### 4.4 Model-based methods

Generative models of the images can be applied to describe the main structural characteristics of visual textures. In model based methods, the estimated parameters of the a priori models assume the role of texture descriptors and can be used for either texture synthesis, classification or segmentation. The most used models in literature are:

– *Autoregressive models*. They assume a direct local interaction between the image pixels, so that pixel intensity is a weighted sum of pixel intensities in a neighbourhood of the pixel and an identically distributed noise. The model parameters are represented by the vector of weights. In a typical texture analysis problem, the parameters are first identified for a given image region by either least square error (LSE) or maximum likelihood estimation (MLE) algorithms, and then used for texture discrimination. For example, in Ref. [59], this approach is exploited to develop a image-guided decision support system able to identify different cases of lymphoproliferative disorders from peripheral blood smears images. In the Local Configuration Pattern (LCP) proposed by Guo and Pietikinen [60], microscopic interactions between image pixels and local shape information are integrated by coupling a linear configuration model with weights determined with LSE optimisation and LBP-based features.
– *Random fields* . Texture can be viewed as a finite sample of a two-dimensional random process that can be described by its statistical parameters. Markov Random Fields (MRFs) are a multidimensional generalisation of the Markov chains, defined in terms of conditional probabilities associated with spatial neighbourhoods. In other words, the probability of a certain cell of a lattice being in a given state (i.e. of a pixel having a specific intensity) is directly determined by the state of neighbouring cells. Hence, texture representation and analysis is translated into a statistical inference problem, where global statistics are expressed in terms of the local neighbourhood potentials. Various formulations of MRFs have been applied to biological texture analysis, especially with the aim of cell and tissue segmentation [61], [62] or cell tracking in time-lapse microscopy [63]. In Ref. [61], texture contextual information is incorporated into an unsupervised binary Markov Random Field segmentation model to automatically detect leucocytes in bone marrow leukemia cell images. In Ref. [62], statistical image modelling of spatial interactions based on Gaussian Markov random fields drives to successful segmentation of cervical tissue images, which is a step towards less expensive cervical pre-cancer detection methods. In Ref. [63], texture-adaptive snakes based on Random Markov Fields are exploited to identify cell trajectories, which is important for the analysis of physiological events in computerized Video Time-lapse Microcopy. The main drawback of these techniques is the computational burden due to the iterative energy optimisation schemes.
– *Fractals*. A fractal is a mathematical concept where a multi-scale set exhibits the same repeating pattern at every scale, which is a paradigm that can be easily transferred to texture analysis. Indeed, fractal parameters can be viewed as a measure of irregularity or heterogeneity of spatial arrangements. Hence, in the last few years there has been growing interest in the application of fractal geometry to observe spatial complexity of natural features at different scales. A number of studies propose inference methods to estimate two main fractal parameters, the *dimension* and the *lacunarity* [64], [65], [66]. These parameters are correlated to texture coarseness (i.e. the larger the fractal dimension and lacunarity, the rougher the texture), and hence can be used as texture descriptors in classification problems where textures are characterised by high irregularity, as in histological images of cancer tissues. Examples of successful application of this approach in recent literature include the accurate classification of cancer cells in breast [67], prostate [68] as well as brain tumours [69].

### 4.5 Transform-based methods

Transform-based texture analysis exploits signal processing techniques to transform the image into a different space, with the aim of highlighting texture properties and maximise the geometrical separability of different types of textures. Texture descriptors are typically inferred from filtered images, on a number of different domains. In the following, we list the most used ones.

**Fig. 6:**
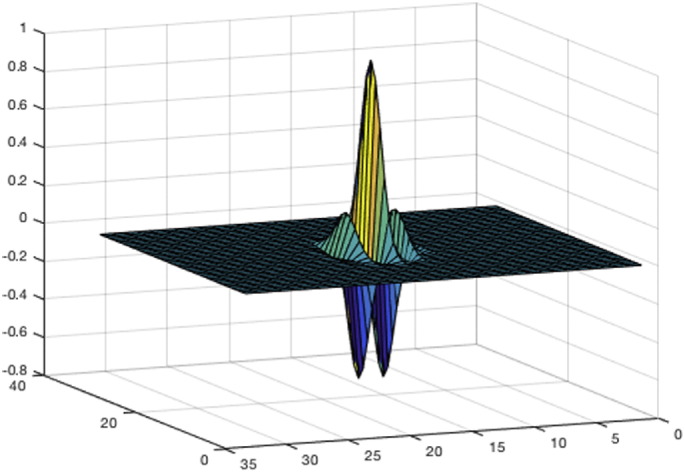
Kernel of a Gabor filter (real part).

– *Spatial domain filters*. Naive spatial-domain methods rely on simple edge detection operators (e.g. Sobel, Roberts and Laplacian filters) and then extract the density of the edges in the filtered image, using it as a texture descriptor. This approach allows to distinguish coarse from fine patterns, but has heavy limitations handling irregular textures, that is the routine in most biological images.
– *Frequency domain filters*. Frequency analysis can be applied, either by means of 2-dimensional Discrete Fourier Transform (DFT) or Discrete Cosine Transforms (DCT), to extract spatial-frequency components of the images. In fact, in the spatial-frequency domain global texture properties such as coarseness, graininess, or repeating patterns can be easily identified. The coefficients of the transforms provide a compact representation of the original image where the most discriminative patterns are emphasised. In literature, they are widely used as texture descriptors, either as they are, or in the form of statistical features or coefficient histograms [70]. However, this approach is generally renowned for suffering from lack of spatial localisation.
– *Gabor and wavelet transforms*. Differently from DCT and DFT, wavelets perform spatial-frequency decompositions where the sinusoidal basis is modulated with different-shaped window functions. The presence of a window with a limited width generally allows much better localisation in the spatial domain compared to traditional Fourier decompositions, ensuring the best discrimination capabilities. On top of that, different window shapes can fit different types of textures. For example, Gabor transform is characterised by a Gaussian-shaped window function (see Fig. 6), which makes it best suited to represent spotted and concentric textures, that are commonly encountered sub-cellular patterns. This trait can be applied to a number of important biological contexts. In Ref. [71], it is exploited to classify 3D immunofluorescence images of HeLa cells, leading to the accurate determination of protein expression changes in response to particular drugs or transgenes. In Ref. [72], it is applied to the detection of sub-cellular changes (e.g. variations of mitochondrial shape) in unstained living cells, which opens the way to the study of programmed cell death (apoptosis) and other fundamental biological processes.

## 5 Feature encoding and dictionary learning

As discussed in the previous sections, several types of descriptors can be used to represent biological textures. Besides quantification, fusing these multiple descriptors into compact and generalisable representations is crucial for boosting the performance of a texture classification system.

The most popular approach for this purpose is the *Bag-of-Features* or *Bag-of-Words* (BoW) model, that was first applied to the context of computer vision in 2009 [73] and then proposed in many variants by the most recent literature, even on biological image analysis. This model takes inspiration from a popular paradigma in text classification, where a bag of words is a sparse vector of occurrence counts of the most representative words in a document. As a parallel of this concept, a *bag of visual words* is defined as a vector of occurrence counts of a vocabulary consisting of local texture features.

**Fig. 7:**
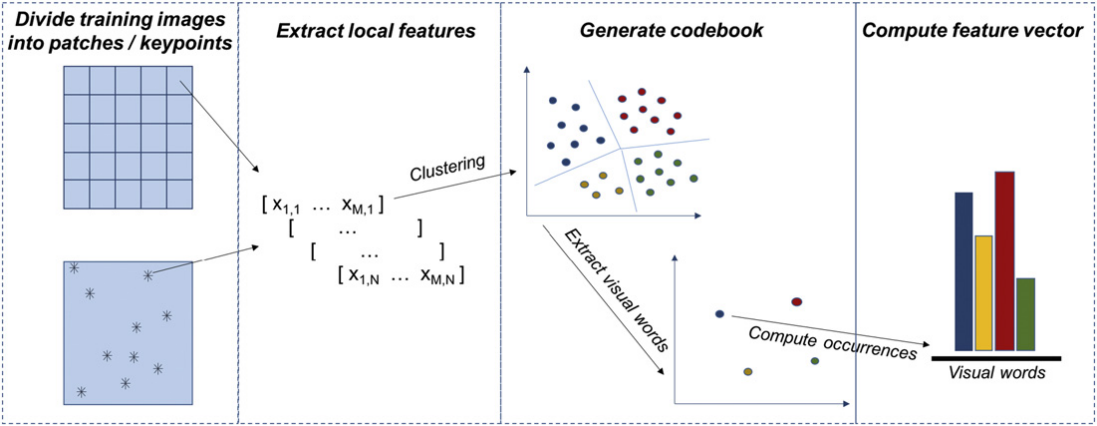
Simplified scheme of BoW feature encoding model.

Fig. 7 shows a simplified representation of the BoW model. First, a large number of local texture features is extracted from the input image (see previous sections). These local features can be either computed from small overlapping patches (e.g. by cropping the image with a regular grid or with a sliding window) or from representative keypoints. A very popular descriptor for this purpose is, for example, the scale-invariant-feature-transform (SIFT), where local gradient information is exploited to extract a large number of keypoints over the full range of scales and locations of the image [74]. As an alternative or in conjunction with SIFT, Speeded Up Robust Features (SURF) can be also computed, that are local descriptors exploiting an integer approximation of the determinant of Hessian blob detector to detect keypoints in the input image [75]. Then, the local features extracted from a representative set of training images are exploited to generate a so-called *codebook*, that is a limited dictionary of elements (the visual words) able to represent in a reduced space all the shared characteristics of the local features from the training set. In the simplest approaches, the generation of the codebook is performed by applying clustering algorithms to the local features (e.g. *k*-means clustering and its variants). By this means, the original *N*-dimensional local feature space is reduced to a *k*-dimensional visual words space, where *k* < *N* is the number of clusters, that is also equal to number of visual words in the codebook. Variants of this approach have also been proposed, where the codebook is learnt by applying either supervised or unsupervised learning techniques (e.g. restricted Boltzmann machines) [76]. Then, the occurrences of the visual features are computed to obtain a feature vector. Another very popular variant is VLAD (Vector of Locally Aggregated Descriptors) encoding, where the codebook is learnt by classical *k*-means clustering, then the residuals of each descriptor with respect to its assigned cluster are accumulated [77].

The step through which the visual features of a novel image are projected onto the codebook elements is called *feature coding*. Depending on the coding function applied to perform the projection, this step can be either performed by hard coding or by soft assignment techniques. After feature coding, a *feature pooling* step (typically based on *sum* or *max* operators) aggregates the projected codes of all the local patches into a single feature vector, which can finally be fed into a classifier to perform texture classification.

In the last few years, BoW framework was extensively applied to the automated categorisation of histopathological images [78], [79]. For example, in Ref, [80] a codebook feature space is created by extracting dense SIFT descriptors at fixed grid locations from a training set of two-photon excitation microscopy images with different stages of liver fibrosis. Then, code vectors are fed into a weighted k-NN classifier to automatically predict the fibrosis stage of unlabelled images.

While traditional BoW model is indeed a major improvement over feature aggregation techniques based on simple concatenation of descriptors, it still suffers from a major limitation, that is the lack of structural discrimination. Indeed, as BoW representation is entirely devoted to representing texture statistically in terms of feature occurrences, any information about object shapes as well as about spatial relations between macro- and micro-structures within the image is completely lost.

As a solution to this problem, recent works apply BoW models coupled with *Spatial Pyramid Matching* (SPM) [81]. This technique performs a hierarchical partitioning of the image with progressively finer level of detail, obtaining at each level an increasingly higher number of sub-images. BoW model is then applied to each sub-image, obtaining a feature histogram pooled over all the coding vectors of such sub-image. Finally, a super-feature histogram is obtained by concatenating all the feature histograms of all the sub-images. This allows to embed the inner spatial relations among sub-images into a compact BoW representation.

Its improved texture discrimination capabilities compared to classical BoW have recently determined the successful application of SPM to the context of cell pattern classification, that is an application requiring fine discriminations of heterogeneous types of textures (see Fig. 3). For this purpose, a variant of SPM called *Cell Pyramid Matching* (CPM) was first proposed in Ref. [82], tailoring the properties of SPM to cell pattern classification. In CPM, each cell image is first resized to a canonical size and then divided into small overlapping patches. To improve spatial discrimination, leveraging on the output of cell segmentation, each cell is also divided into an inner region, which covers the cell content, and an outer region, containing information related to cell edges and shape. The patches are then represented by patch-level features based on SIFT and DCT descriptors. The local histogram from each patch is extracted by using a pre-trained visual word dictionary, and the local histograms of each region are pooled to compute the overall histogram of that region. Finally, the cell image is represented by the concatenation of the regional histograms. More recently, a two-level cell pyramid was used in a similar fashion also by Manivannan et al. [83] to capture spatial structure within immunofluorescent HEp-2 cells, leading to highly accurate diagnosis of autoimmune diseases.

## 6 Latest trends: self-learnt features and deep learning models

All the works and techniques reviewed so far have a common trait, in that they are all based either on handcrafted image descriptors or on some predefined models of texture. As such, the discriminative capabilities of each technique depend on (i) how faithful the model is to the actual characteristics of the images to be analysed; (ii) how efficient the descriptor is in terms of compactness as well as of robustness to image variations, when it is fed into an automated classifier. Hence, the general focus of the last decade's research has been on the design of texture representation schemes embedding these two concepts.

However, this approach has two limitations. First, it requires deep a-priori knowledge of the characteristics of the textures that have to be analysed/segmented/classified. This is possible for artificial textures, but not so easy with natural textures, and even more difficult when the texture is triggered by a biological reaction that is driven by mostly unknown mechanisms. Second, it is strongly application-dependent. That is to say, any texture model is at its best when the imaging conditions are very limited and constrained. Hence, a texture descriptor that is perfectly suited for a specific category of images does not ensure the same performance when it is applied to a different type of images.

Based upon these observations, the latest trend is to abandon the design of handcrafted features, and let the texture analysis framework learn the model directly from the images. The research community agrees that deep learning (DL) has the highest potential in this scenario [84], [85], [86].

In recent years, DL architectures have become more and more popular in many sectors of computer vision and pattern recognition. These methods are essentially based on distributed representations of the information, with the underlying assumption that the observed data can be represented by interactions between multiple punctual factors, organised in layers. Each layer corresponds to a different level of abstraction, on a hierarchical basis from the lowest to highest: the former conveys more low-level information about the distribution of pixel intensities, while the latter provides a more abstract representation of the input. Hence, the level of abstraction can be easily modulated by varying the number and size of the layers.

**Fig. 8:**
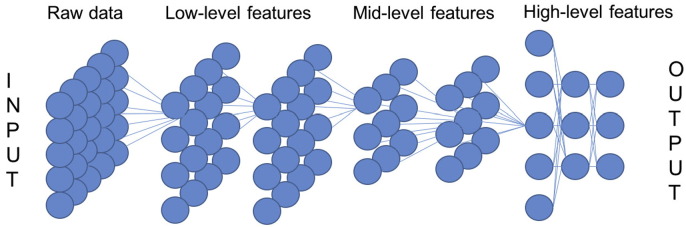
Deep neural network framework.

An image can fed into a deep learning network in its raw form, as a vector of pixel values. Each layer is locally connected to the previous one, and learns features that can be extrapolated to describe the texture of the input image at progressively higher levels of abstraction, typically exploiting the backpropagation algorithm (see Fig. 8). A first layer usually provides a map of the edges in the image at specific locations or at specific orientations. A second layer performs rough pattern detection, in that it detects particular arrangements of the edges. A third layer might detect spatial combinations of such patterns, and so on. Hence, deep learning architectures can be exploited to obtain compact and non-redundant intermediate representations of textures, obviating the extraction of handcrafted features [84].

Many deep learning algorithms can be applied to either supervised or unsupervised tasks. A detailed analysis of these algorithms is out of the scope of this paper. In this section, we will give just a glimpse into few of the most popular deep learning techniques in the context of texture analysis, with special regard to biological imaging applications.

Most deep learning applications typically use feedforward neural networks, where the network learns to map a fixed-size input (e.g. the raw image) to a fixed-size output (e.g. a label, or a probability of belonging to a specific texture category).

**Fig. 9:**
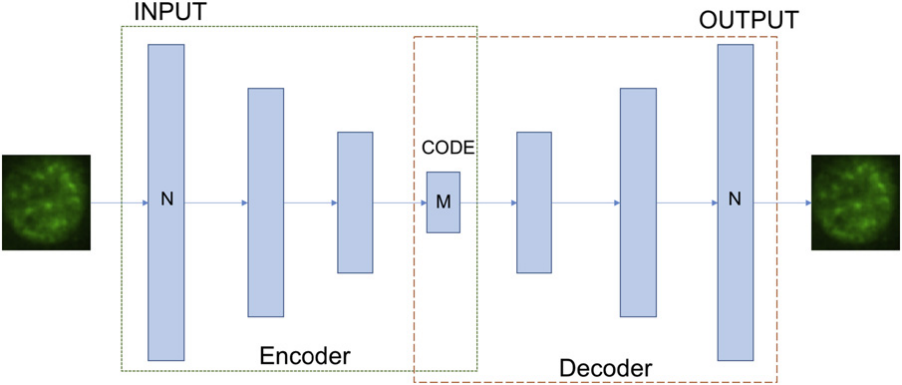
Structure of a deep autoencoder with 5 hidden layers.

A popular architecture in this context is the deep *Autoencoder*, a simple unsupervised network mapping the input to the output through backpropagation algorithm. The aim is reproducing the input with the least amount of distortion possible (see schematic representation in Fig. 9). The architecture is composed of two, symmetrical deep-belief networks, that respectively represent the encoding and the decoding half of the net. The encoding layers produce a compressed representation of the input, with progressively higher level of feature size reduction. The more the hidden layers, the higher the level of size compression. The decoding layers reconstruct the input at its original feature size. Hence, the intermediate layer (*code*, in Fig. 9) provides a reduced set of representative features that can be used for biological texture classification problems.

For example, autoencoders were successfully used to perform nuclei detection on high-resolution histopathological images of breast cancer. In a recent work by Xu et al. [96], the autoencoder learns high-level features from raw pixel intensities to identify distinguishing textures of the nuclei. Image patches represented by the autoencoder's high-level features are subsequently fed into a classifier which categorises each patch as nuclear or non-nuclear.

Inspired by the multi-stage processes in the visual cortex, in the very last period supervised approaches based on*Convolutional Neural Networks* (CNNs) have emerged as the state-of-the-art deep networks. A typical CNN architecture contains a number of convolutional layers interlaced with subsampling layers (respectively devoted to feature extraction and pooling), followed by fully-connected layers devoted to classification. The key to the success of CNNs is the ability to learn increasingly complex transformations of the input and capture invariances from large labelled datasets. This makes this deep network particularly suited to handle heterogeneous textures. On top of that, CNNs have shown promising results in the emerging topic of *domain transfer*, where large image datasets are exploited to obtain pre-trained general-purpose texture feature extractors, that can be transferred to other domains of biological imaging [97].

Hence, in recent years CNNs is becoming increasingly popular in the field of biological texture analysis, with several important applications including mitosis detection in histology images [98], [99], [100] and the classification and grading of cancer cells [101], [102]. The most important drawback in this case is the need for very large datasets to learn representative features, which is currently limiting a broader applicability of this very promising technique.

For better positioning deep learning techniques (and CNN in particular) in the panorama of biological texture analysis, we chose to show as a case-study the outcome of the most recent contest on fluorescence HEp-2 cell pattern classification hosted by ICPR, which is one of the most reputed conferences on pattern recognition [90]. This case-study was chosen for two main reasons. First, because the accuracy results are completely unbiased, as they were computed based on one-image-out cross-validation by a third party (i.e. the organisers of the contest) on a testing dataset that was at that time unavailable to the participants. Second, because the competition had been repeated three times since 2012, obtaining a very good participation rate. This makes it a significant case-study not to merely rank the individual descriptors (which would be anyway limited to the context of HEp-2 classification), but rather to analyse the general trends of the proposed research contributions, that is a concept that can be generalised to other imaging applications.

While the participants of previous editions of the contest had focused on identifying the best texture descriptors per se (e.g. improved formulations of local binary patterns [22]), in the latest edition most of the research groups directed their efforts to designing more effective feature encoding techniques (such as CPM or other BoW variants [23], [87], [88]). This suggests that the sophisticated aggregation of different types of multi-scale descriptors by means of feature encoding techniques is the state of the art at the moment. Only one out of the eleven participants proposed a deep learning approach, based on CNN [89] (highlighted in grey, in Table 1). This, again, is not surprising, as deep networks are quite consolidated in other fields of pattern recognition, but not much explored in the context of texture analysis. Quite notably given the limited size of the training set, which is a well-known drawback of deep learning, CNNs performed comparably with the well-established approaches [90].

As the attractiveness of deep learning architectures is rapidly growing, recent literature presents many more applications to biological texture analysis with encouraging results. For example, in Ref. [103] a combination of hand-crafted features and features learned through CNNs were applied to mitotic cells detection and counting for breast cancer grading. In Ref. [104], deep learnt features applied to the detection of basal-cell carcinomas were shown to outperform pre-defined bag of words representations. Finally, an increasing number of recent works successfully applied deep CNNs to nucleus detection and classification, which is one of major tasks of histological image analysis. Among the others, Xie et al. [105] recently proposed structural regression CNNs to learn a proximity map of the cell nuclei, while Sirinukunwattana et al. [106] applied a Spatially Constrained variant of Convolutional Neural Networks (SC-CNN) to nucleus detection and classification in colon adenocarcinomas. Unlike previous works based on traditional texture analysis, these approaches have the major advantage of not requiring a preventive segmentation of the nuclei.

## 7 Summary and outlook

Texture analysis is an important research topic in biological imaging, because it allows the characterisation of subtle properties of cells and tissues that cannot otherwise be easily quantified. As such, the most successful techniques proposed by literature are the ones able to cope with the inherent variability and noise of biological textures. This can be obtained either by redesigning descriptors borrowed from other computer vision applications, or by applying sophisticated feature encoding techniques to condense different types of local information into compact, multi-scale and invariant texture representations.

Besides approaches based on the extraction and encoding of handcrafted texture descriptors, the latest trend is to apply deep learning architectures, that can learn the texture model directly from the images. In spite of its shortcomings (first of all, the necessity of very large image sets), it is very reasonable to think that deep learning will be attracting more and more attention in the near future, as its full potentials in the context of biological texture analysis are yet to be discovered.

## Figures and Tables

**Table 1:** Results of the *Performance evaluation of indirect immunofluorescence image analysis systems* contest.

Ref.	Textural features	Classifier	Accuracy
Mannivannan [23]	Four types of local features with CPM-BoW encoding	Ensemble SVMs	87.09%
Sansone [87]	Dense local descriptors with BoW encoding	SVM	83.64%
Theodorakopoulos [88]	SIFT with VLAD encoding, LBP-based and morphological descriptors	SVM	83.33%
Gao [89]	Raw image data with deep CNNs	Deep CNNs	83.23%
Paisitkriangkrai [90]	Combination of different sets of low-level texture features	Boosting classifier	81.55%
Ensafi [91]	SIFT and SURF descriptors with BoW sparse encoding	SVM	80.81%
Nanni [92]	LBP-derived and morphological features	SVM	78.27%
Codrescu [93]	Raw image data	Neural networks	74.93%
Taormina [94]	Combination of different types of local texture features	kNN	74.62%
Ponomarev [95]	Morphological and shape descriptors	SVM	73.53%
Roberts [90]	Wavelet transform-based features	SVM	66.99%
